# Reflections on Darwinian Evolution – Is there a Jewish Perspective?

**DOI:** 10.5041/RMMJ.10044

**Published:** 2011-04-30

**Authors:** Chaim O. Jacob

**Affiliations:** Professor of Medicine and Molecular Microbiology & Immunology, The Keck School of Medicine, University of Southern California (USC), USA; Director of the Federation of Clinical Immunology Societies (FOCIS) Center for Excellence at USC, Los Angeles, CA, USA

**Keywords:** Evolution, fossil, Judaism, religion and science, Moses Maimonides

## Abstract

I present a realistic view of what Darwinian evolution is in its current form and what it is not. I argue that the Torah is not a source of scientific knowledge and all attempts to reconcile its plain text with the data of science are an exercise in futility. The article argues the position that science and the Torah are incommensurable. I argue against using the Torah for attaining knowledge about the nature of the world, or using science for enhancing or denying the truth of the Torah.

## INTRODUCTION

As part of the 150th anniversary of the publication of *On the Origin of Species*, a prominent Orthodox Jewish physician and ethicist has published in *RMMJ* a comparative analysis between what he calls “the scientific aspects of the theory of evolution and a Judaic approach to these aspects”.^1^ But rather Steinberg presents a creationist fundamentalist view masquerading as rational and “reasonable debate ... in a calm and humble way”. In his essay, Steinberg digresses into discussing some issues concerning the science–religion debate that are clearly irrelevant to evolutionary theory. His arguments are partly misleading and mostly incorrect.

One is tempted to let it go and pass indulgently and in silence over this entanglement into whitewashing apologetics. But Maimonides in his *Guide of the Perplexed*^2^ holds the opinion that it is better to bring no proof at all in favor of the Torah than to bring a poor proof, because a poor proof brings the whole system under suspicion; no proof does not. So as a Jew deeply committed to Halakhic Judaism and as practicing geneticist, I cannot refrain from offering some reflections in order to rectify the false picture presented on both subjects, Darwinian evolution and the Jewish perspective.

## SCIENCE AND DARWINIAN EVOLUTION

Steinberg states that “Judaism accepts all experimentally proven facts and observations of the theory of evolution ... but rejects other assumptions and speculations which contradict fundamental Jewish beliefs, and which are anyway not scientifically proven”. He presents a widely, but incorrectly, believed perception that the basis of all scientific knowledge is facts, which are obtained by experiments and observations. Accordingly, science begins with facts – observations about nature that can be verified by other scientists. Only after an agreed-upon body of facts exists can one begin to formulate theoretical concepts that might explain them.^1^ However, this view is wrong: science does not begin with facts; rather, all experimentation begins with the premise “Let us assume that...”. In short, science starts with theories and concepts about the physical world. Only after a theoretical framework has been formulated can one understand what the facts are. Boldly put, “facts are ventriloquists’ dummies. Sitting on a wise man’s knee they may be made to utter words of wisdom; elsewhere, they say nothing, or talk nonsense, or indulge in sheer diabolism” (quote attributed to Aldous Huxley). Science advances by postulating concepts and making assumptions, and then investigating to determine whether these concepts and assumptions are successful in explaining and predicting phenomena observed in nature or in the laboratory. As successful explanations grow, it becomes more and more plausible that the assumed concepts and ideas are basically correct and become scientific “facts”. In science, “fact” can only mean confirmed to such a degree that it would be perverse to withhold assent. I suppose it is possible that the theory of gravitation is false and apples might stop falling to the ground and start rising, but the possibility does not merit any serious consideration.

Steinberg further states that “it is important to distinguish between conclusions drawn from controlled experiments, and a theory, a speculation, or an assumption”. Consequently he charges that many of the premises of the evolution theory are unproved speculation: “evolution is only a theory; therefore, one can accept that which is fact and experimentally proven and reject that which is an unsubstantiated hypothesis, or replace it by an alternative explanation”. It is important to emphasize here that facts and theories are not rungs in a hierarchy of increasing certainty. Facts are the world’s data, while theories are structures that explain and interpret data. Facts do not go away when scientists debate rival theories to explain them. Einstein’s theory of gravitation replaces Newton’s, but apples did not suspend themselves in mid air, pending the outcome. Humans evolved from apelike ancestors whether they did so by Darwin’s proposed mechanism or by some other yet to be discovered.^3^

Steinberg introduces a list of creationist claims but without being able to bring one single reference from the professional peer-reviewed literature, because creationist standards of scholarship are too low for publication in professional, reputable journals. For example, he challenges the fossil evidence, including the claim of a “missing link”, namely the lack of transitional fossils from lower to higher species.

Even a cursory analysis of the professional literature would prove that these claims are incorrect. Based upon the consensus of numerous phylogenetic analyses, the chimpanzee is the closest living relative of humans. Thus, we expect that organisms lived in the past which were intermediate in morphology between humans and chimpanzees. Indeed, over the past century, many spectacular paleontological finds have identified such transitional hominid fossils. Or take the Ambulocetus (the “walking whale”), the transitional fossil that shows how whales evolved from landliving mammals. As S. J. Gould puts it, “If you had given me a blank piece of paper and a blank check, I could not have drawn you a theoretical intermediate any better or more convincing than Ambulocetus. Those dogmatists who by verbal trickery can make white black, and black white, will never be convinced of anything, but Ambulocetus is the very animal that they proclaimed impossible in theory.”^4^

Continuing, Steinberg writes that “one of the most challenging problems of the theory of evolution is the origin of life” and that Darwinian evolution fails to explain how life arose and developed. To put it mildly, this is a rather odd statement for a biologist. It does not take an expert to know that evolution theory is not about “how life arose”. Evolution theory is about the evolution of the variety of living organisms from a common ancestor. As to the origin of that common ancestor, the first replicator, this question is beyond evolution theory. The essence of the theory of evolution is that organisms are related by descent from common ancestors. Over time, organisms change and diversify as they adapt to different environments. Species that share a recent common ancestor are more similar to each other than species whose last common ancestor is more remote. Thus, humans and chimpanzees are, in configuration and genetic make-up, more similar to each other than they are to baboons, elephants, or kangaroos. But other concepts, commonly used in literature about Darwinian evolution (especially in popular literature), such as “survival of the fittest” should not be understood simplistically and to a large degree are not essential to the modern understanding of Darwinian evolution. In fact most currently available information leads us away from the idea of survival of the fittest and toward a model of survival of the “barely tolerable”.^5^

Tending to oversimplify the concept of “survival of the fittest”, we might expect that the most impressive results of evolution are the complex and perfected adaptations of organisms to their environments. For example, there are those that propose that the capacity of each individual to mount an immune response to a pathogen represents human evolution in miniature.^6^–^8^

There is enormous variation and diversity in the antibody population – the system is capable of recognizing more than 10^8^ antigen patterns. By recombination, mutation, insertion, and deletion, gene fragments are packaged by lymphocytes, forming populations of receptor complexes that compete to take hold of foreign antigens. Those that succeed get to reproduce their progeny. The successive rounds of mutations and selections that occur allow the body’s immune system to choose a population of cells that specifically synthesize the correct antibody profile to combat the specific infection. The truth of the matter, however, is that no evidence for evolution is stronger than the presence of rudimentary or vestigial structures in nearly all organisms including humans. “Remnants of the evolutionary past that don’t make sense in the present – the useless, the odd, the peculiar, the incongruous – are the signs of Darwinian evolution”.^9^ Indeed, the immune system demonstrates evolution, but not because it has perfected adaptation of the antibody molecule to the specific infectious agent, but rather because it is clumsy and built from odd parts. As a defense organization, the immune system is large, complicated, and wasteful; it is slow to react and fights today’s threats with the solutions of the past.^10^ The so-called opponents of the immune system – viruses, bacteria, and parasites – are hardly predictable and are rapidly changing, so past experience does not necessarily prepare the host’s immune system for future challenges. While the selective forces acting upon the immune system are constantly varying, the products of the immune cells are often poorly adapted to a particular set of circumstances. Consequently, there is a continuing loss of life from infectious diseases.

When new features evolve in a species, they tend to build on already existing features. They are not built from scratch.^11^ (Francois Jacob elaborated a model of evolution as “tinkering”. According to Jacob, natural selection only works with the materials available and within the constraints present at a particular time in a particular place.^11^) From an evolutionary standpoint new features do not need to have the best possible design. They just need to be good enough to allow the organism to live long enough to reproduce. The evolution of the human body is no exception. We have body parts whose design is deficient, but they have been tolerable enough to keep our species from extinction. Let us consider the following suboptimal designs in the human body: The human pharynx is the part of the throat that begins behind the nose and leads down to the voice box. It does double duty as a tube for breathing and for swallowing. But when you are swallowing you cannot breathe, and when you are breathing you cannot swallow. That is why humans run a serious risk of choking if the pharynx does not close at the right time when eating. Curiously, human infants under 6 months and chimpanzees do not have this problem. But infants and chimpanzees cannot talk, and without our uniquely situated pharynx we would not be able to talk either.

The evolutionary innovation of bipedalism – walking upright on two legs – forced a smaller pelvis on us. But bipedalism is not the whole story. Humans have evolved big brains, and big brains needed big containers to hold them. This is why human infants are born more premature and helpless than other mammals. Babies need to get through the birth canal before their heads get too big. The small birth canal is responsible for significant death of mothers and infants during the complex process of birth.

Compared to our *Homo erectus* ancestors who had massive jaws with huge molars, the human jaw is too small for the number and size of our teeth. Many people have no room for wisdom teeth (third molars) if they get them, and a lot of people’s teeth have to fight one another for limited space, leading to crooked teeth. Impacted wisdom teeth can result in serious infections, and before modern dentistry these late emptors could be deadly. These facts may be related to an inactivating mutation in a myosin gene (*MYH16*), a chief component of the powerful jaw muscles of many non-human primates who share large crests on their skulls to which their heavy jaw muscles attach. All modern humans share a defect in the gene that created this protein, which could have left us unable to produce one of the main proteins in primate jaw muscles, and as a consequence the crest on the skull for the muscle attachment is notably absent from all modern human skulls.^12^ Our ancestors may have lost their skull crests when our jaw muscles stopped exerting so much strain on the skull. By doing away with large anchors for chewing muscles, our skull may have freed itself to grow into its modern, rounded shape, and unconstrained our brain to increase its size.

The human vermiform appendix, a 5–10 cm long and 0.5–1 cm wide pouch that extends from the cecum of the large bowel, is a derivative of the end of the phylogenetically primitive herbivorous cecum found in our primate ancestors.^13^ The human appendix has lost its previously essential function as a cellulose-fermenting/digesting cecum and has no apparent function in modern human. Indeed, people who completely lack appendix from birth have no apparent physiological detriment, and appendectomy is without currently discernible long-term side-effects.^14^ Since evolution is not keen on cleaning up after itself, we are left with a potentially life-threatening situation when indigestible food that enters the appendix is not forced out by muscular contractions. In as much as 7% of the population in industrialized countries, the appendix becomes inflamed and must be surgically removed to avoid a critical infection.

## THE “JEWISH FAITH” AND THE THEORY OF EVOLUTION

Steinberg writes: “Judaism, as a monotheistic religion places an absolute truth in the existence of an Almighty God ... who created the world, established the rules of nature, and commanded a moral-religious practice embodied in the Bible which was given to the Children of Israel on Mount Sinai around 3,300 years ago (around 1290 BC)”. In contrast “science has inherent limits ... it is constantly altered and changed as new discoveries and facts develop. The mere fact that a scientific theory is accepted by the majority of scientists does not prove that it is correct ... [T]he theory of evolution, which at first may be widely accepted ... may be [later] proven to be partially or totally incorrect”.^1^

I hold the opinion that it is ill advised and wrong to attempt to protect the truth of the Torah by casting doubt on the certainty of scientific understandings and/or by trying to prove that scientific truth is not absolute but rather inconclusive or preliminary. Such strategy mistakenly treats the Torah as a text-book in physics, chemistry, or biology, as if the *Shekhina* had descended onto Mount Sinai to fulfill the functions of a university professor. Hence it is wrong to regard revelation as a substitute or supplement for natural knowledge or consider Scriptures a body of information on the nature of the world or its history.^15^ One should be aware of the paradox evoked when one regards the Torah as superior only to the extent that the information provided to him is more reliable than his biology book. Information obtained from historical, physical, or biological inquiry that satisfy human curiosity should be deemed within the realm of the profane. If the “Holy Scriptures” were a source of information, it would be problematic to see their sacredness. Therefore, any literal reading of the first chapters of Genesis is misguided, whether to show that the Torah is wrong (evolution is a process of millions of years rather than six days) or when modern science is used to validate the Torah (such as the assertion of the biblical statement “and there was light” by the “scientific discovery that the universe began with the sudden appearance of an enormous ‘ball of light’ ... dubbed ‘the big bang’”).^16^

It is arrogant for us to determine that we are at the center of a 5,770-year-old world, capable of understanding all of “divine” creation from words written in a few paragraphs in the book of Genesis. Nevertheless, Steinberg is, of course, entitled to his fundamentalist position regarding how to read the Torah. Conversely, one cannot accept the claim that his perspective is the unified view of Halakhic Judaism. In fact, throughout his article, Steinberg considers Judaism as representing a significant unity of beliefs which includes a particular conception of man, of the world, or of its history. This view is clearly erroneous as any analysis of Jewish intellectual history can prove.

Jewish doctrines and principles were so diverse and dependent upon different schools of thought pertinent to their epoch that they can hardly be alleged to present any significant unity.

Hence, Judaism as a historical entity was not constituted by its set of beliefs or philosophical opinions. (I argue that the core of the Rabbinic Judaism is its religious practice determined by the Halakha. However, I do not argue that there is no system of belief behind its practice, but instead that it is not intended to be a picture of the world. It is just a framework in which one conducts practices that are supposed to be appropriate. See also Jacob.^17^) In truth, articles of faith were the subject of fierce dispute throughout Jewish intellectual history. Even the interpretation of the idea of divine unity by rabbinic thinkers is characterized by direct oppositions. The primary document of Jewish faith, the *Shema,* opens with the verse “Hear O Israel, the Lord our God, the Lord is One”. We should pay attention that the “One” of Isaac Luria (1534–1572) is incompatible with the “One” of Maimonides. We should be acutely aware how dangerously close the Jewish Kabbalists’ belief in a decimalian system of deity is to the Christian Trinitarianism. Therefore, Steinberg’s assertion that “it is a cardinal axiom of Judaism that God created the world from nothing”^1^ is simply incorrect. The presence of Rabbi Levi ben Gershon’s (Gersonides) biblical commentary in the rabbinic *Miqraot Gedolot* Bible is a wonderful testimony to the openness of the rabbinic tradition to diverse theological interpretations, in total difference to the picture presented by Steinberg. Gersonides’ (1288–1344) radical hermeneutics is expressed not merely in seeing the biblical account of creation in non-literal terms – that is not unusual for the rabbis – but in applying a philosophical interpretation to that event which both limits and depersonalizes God and feels compelled to reject the notion of creation *ex nihilo.* Creation for Gersonides is an event in which God functions as the donor formarum (*noten ha-shurot*). While Gersonides is convinced that creation in all its parts testifies to God’s beneficent design, the creator is yet constrained to work with that which is co-eternal with him.^18^

Although Maimonides’ opinion on this issue is far from being clear, in the *Guide of the Perplexed* he states: “We do not reject the Eternity of the Universe, because certain passages in Scripture confirm the Creation; for such passages are not more numerous than those in which God is represented as a corporeal being; nor is it impossible or difficult to find for them a suitable interpretation. We might have explained them in the same manner as we did in respect to the Incorpo-reality of God. We should perhaps have had an easier task in showing that the Scriptural passages referred to are in harmony with the theory of the Eternity of the Universe if we accepted the latter, than we had in explaining the anthropomorphisms in the Bible when we rejected the idea that God is corporeal...”.^19^ Thus, in principle, believing in creation from pre-existing matter is not incompatible with the Torah.

More importantly, however, for the purpose of my argument here, Maimonides’ or Gersonides’ specific opinions on this issue are irrelevant. They are men of the Middle Ages, and their scientific views are deeply rooted in their times. Dr Steinberg and I, however, are men of the twenty-first century, and when we talk about “science” we should refer to what we mean by science today and not to what they represented in the Middle Ages. To us, the importance of chapter II:25 of the *Guide*^19^ rests in the approach of Maimonides to contradictions between the literal meaning of a Torah verse and well established knowledge, say, modern science. In such a case, Maimonides affirms that one should accept the science, reject the literal meaning of the Torah verse, and understand the verse figuratively: “For if the Creation had been demonstrated by proof, even if only according to the Platonic hypothesis, all arguments of the philosophers against us would be of no avail. If, on the other hand, Aristotle had a proof for his theory, the whole teaching of Scripture would be rejected, and we should be forced to other opinions. I have thus shown that all depends on this question.”

There is, indeed, a clear and extensive history to claims that the scientific knowledge of the rabbis of the Talmud was based on the theories current in their time and can be disproven by later scientific discoveries. For example, the Mishnah mentions the existence of a mouse that was half animal and half dirt.^20^ Since the sages obviously did not witness this imaginary creature themselves, they, probably, either read about it (perhaps in Plinius’ *History of Nature* 9:58) or heard about it from others. Similarly, the Talmud seems to accept that the human heart has only two chambers.^21^ This was indeed in accordance with how Hippocrates and Galen understood the heart at the time. Maimonides explicitly states, with respect to these very issues, that they are outside the limits of acceptable rabbinic authority: “Do not ask of me to show that everything they have said concerning astronomical matters conforms to the way things really are. For at that time mathematics were imperfect. They did not speak about this as transmitters of dicta of the prophets, but rather because in those times they were men of knowledge in these fields or because they had heard these dicta from the men of knowledge who lived in those times.”^22^

But Steinberg argues the exact opposite: the rabbis of the Talmud had divine assistance in understanding scientific reality. So if contemporary science disagrees with the sages’ perception of reality, then evidently nature has changed. Hence, Steinberg claims that intraspecies changes, “micro-evolution”, have been demonstrated and “indeed, already early rabbinic authorities described numerous intraspecies changes between the Talmudic period and their own”.^1^ They call it “Nature has changed”, and Steinberg enumerates them in his Encyclopedia.^23^ I am deeply puzzled: Is this an error effecting the naive, or perhaps a pretense of naïveté, claiming the existence of a mouse that was half animal and half dirt or a two-chambered heart which has changed in the evolutionarily minuscule time-period of 1,500–2,000 years?

Indeed changes in climate, diet, hygiene, and accessibility of clean water and food have caused biological relevant changes in human life expectancy, average height, and time of appearance of menstrual cycles in girls, as has been amply demonstrated scientifically. But the laws of nature have not changed: living creatures can arise only from other living things. I wonder why the same scientific standards Steinberg keenly demands from evolutionary biologists are not applied to those rabbinical claims that nature has changed. If, indeed, one would search for demonstrable proof to the validity of any claim that “nature has changed” in rabbinic literature, regrettably one would find absolutely no such evidence.

It seems that Steinberg pays only lip-service to the transcendental position in Judaism that became an essential part of Jewish theology since the Middle Ages to these days. I am puzzled by the obsession to locate a transcendental deity in the middle of the debate over how the universe came into being, whether the universe is eternal or created at a certain time, and how, when, and what is its history. It seems that he is not aware, or rather chose to ignore, the considerable theological challenge this view produces. By accepting an unconditional transcendental God, one must dismiss any notion of ontological reality, namely, the assertion of Godly cosmic intelligence which is reflected in the world and its functions. All knowledge, no matter where, how, and by whom it is produced, ought to be discussed unrelated to an ontological reality (of which we know nothing and cannot know anything). It should be emphasized that the transcendental position in Judaism did not start with the Jewish philosophers of the Middle Ages; evidence for this position can be found among Chazal in the Talmud; for example, Babylonian Talmud.^24^

Interestingly, some of our contemporary Orthodox scientists and rabbis have revived the medieval scholastic argument (which is Christian in its origin) that there is no necessary conflict between science and religious belief since God wrote two books, the Bible and the “Book of Nature”, by which his existence and intentions could be known. Therefore, the study of nature had religious value, and the notion that humans should use their God-given faculties of observation and reason to read the “Book of Nature” accurately could be regarded as a religious duty.^6^,^25^

I strongly disagree with this view, and I am acutely aware of its consequences. We must not deceive ourselves into believing that the Torah provides any more useful information regarding nature than the natural sciences provide about the Torah. Invoking this old idea is not only problematic from the perspective of Halakhic Judaism, but it also reflects a deep misinterpretation of current natural sciences, as amply exemplified by Steinberg’s article. There is a decisive difference between what was called “science” in ancient and medieval times and what is called “science” today, and Steinberg seems not to pay attention to it. The major change that took place in the scientific outlook (starting roughly in the seventeenth century) was the introduction of the concept of the functional relations among the phenomena investigated by science. Modern science succeeds by looking solely for functional relations across factual data. Experimental biology, as physics beforehand, refrains from dealing with problems of life itself and focuses upon its active mechanisms. These mechanisms are described by the functional relations among phenomena. The question remains purposefully open whether these mechanisms, being described by biologists, constitute life itself or are no more than mechanisms active in life. Indeed, Claude Bernard makes a distinction between the essence of life and the mechanisms acting in life.^26^ In my opinion this distinction is valid today, as it was 145 years ago. Generally speaking, what used to be called science until modern times did not differentiate between the mechanisms functioning in the world and the essence of the world itself. Nature was understood as expressing a purpose, meaning, or value embodied in the phenomena. The conception of nature and the world in terms of meaning dictated the way people looked on natural data. For example, the Aristotelian-Ptolemaic conception of the celestial bodies revolving with uniform velocities in circular orbits was not derived from observation. In fact, observations suggested all these movements to be neither circular nor uniform, but because circular and uniform movement was deemed as the perfect movement, and nature was supposed to express this meaning, an astronomy was devised in order to comply with the paramount notion of a perfect and meaningful universe.

This is the background for the long-standing confrontation between “religion” and “science”. If the content and conclusions about natural phenomena bear specific meanings and are expressions of these values, then matters of science are on the same plane as matters of faith, namely both enterprises deal with questions of meaning. If this view is accepted, then religion and science may be intertwined, mutually antagonistic, or supplementary as the case may be. However, today we have no “science” in the sense of the Middle Ages, in which religion and science meet. Modern science and the Torah are entirely alien to each other.

Since natural sciences have gradually and progressively liberated themselves from the task of discovering the meaning of reality and become exclusively interested in functional relationships, they have become indifferent to any and all issues of meaning, purpose, or value (an important qualification: the practice of medicine is a problematic discipline in relation to other sciences, in that its practice has major moral and value consequences and therefore should not be considered in the present discussion). This is the only context in which natural science is objective; it is uniform and common to all who understand it and is independent of the varying outlooks and values of those involved in scientific discourse. While the scientific discourse should be (by definition) understandable to anybody who acquires the knowledge of its language, the religious discourse, the language of the Torah is infinitely “incomprehensible” and needs constant interpretation. “The Torah speaks in the language of man” – we should always recognize that we are talking only in metaphors (This expression frequently arises in the Talmud as representing Rabbi Ishmael’s approach.^27^ Rabbi Ishmael means to say that the Torah contains certain verses that should be taken in the plain sense and not expounded homiletically. However, I use this remark in accordance with Maimonides’ interpretation, namely this expression implies that the Torah employs language that is suited to the understanding of the masses, and therefore one should not take the Torah’s words at face value. See, for example, *Guide of the Perplexed:* “You, no doubt, know the Talmudic saying, which includes in itself all the various kinds of interpretation connected with our subject. It runs thus: ‘The Torah speaks according to the language of man’, that is to say, expressions, which can easily be comprehended and understood by all, are applied to the Creator. Hence the description of God by attributes implying corporeality, in order to express His existence: because the multitudes of people do not easily conceive existence unless in connection with a body, and that which is not a body nor connected with a body has for them no existence.”^28^) – since the language of man is incapable of expressing divine matters. Leibowitz expressed this idea colorfully: “No expressions in ordinary language are adequate for speaking of God and of the position of mankind before God. Utterances of divine matters require careful scrutiny if one is to distinguish intended sense from literal meaning. Words may seem simple and unambiguous, such as ‘and God descended upon Mount Sinai’. Yet most of us understand that God does not dwell on the top of a cosmic skyscraper from which he descends in a helicopter. The same applies to all that is said in the so-called ‘historical books’ of the Bible”.^29^

Those of us who accept Halakhic Judaism acknowledge that Torah texts are unchangeable, and their study deemed the very highest of religious work. However, none of the readings and understanding thereby produced are or should be considered final. Thus, if experience appears to contradict an accepted interpretation of a text, we should search for a new interpretation, rather than denying the authenticity of our knowledge. We are indeed constituted by our books but categorically not by a single way in which these books can be read or understood.

As a practicing scientist and an educated member of society, I subscribe to the notion that the best way to achieve knowledge about the world and the processes acting within nature is by applying the scientific method. The accomplishments of science in terms of conclusions, deductions, and inferences are not dependent on a person’s willingness to accept or reject them but rather are forced upon those that know them. Thus, one has no free will to accept or reject the scientific truth of Darwinian evolution. Scientific research determines the truth (I am referring here to scientific truth which is instrumental and synonymous with “according with scientific method”, to differentiate it from “truth” as a value, which is not imposed, and which one can ignore even if he knows for a fact that it is the truth) about reality even against the will of the scientist. In fact, the scientific truth imposes itself upon the investigator if he wants to achieve any theoretical or practical result. Intentional deceit or falsification is usually detected because the scientist’s work is open to the critical scrutiny of his colleagues. Although continuation of the scientific activity may reveal in the future a somewhat different picture of reality, adherence to the scientific method is the only option that will allow us to rectify with time our mistaken scientific concepts.

In absolute contrast to the scientist in me, I am, at least to a certain degree, acting as a free agent when it comes to the practice of Judaism. To my knowledge, the choice to put on phylacteries this morning had practically nothing to do with whether I have irrefutable evidence to the existence of God, the creation of the world, or whether the biology I am studying the rest of the day enforces or denies my religious convictions.

While the position for which I argued here is that science and the Torah are incommensurable, there is one aspect in which Torah scholars and scientists are exactly in the same situation. Rabbi Naftali Zvi Yehuda Berlin (1813–1893), the *Naziv* in his introduction to his *Ha’amek Davar,* explains why he felt the need to write a new commentary on the Torah (my own translation): “just as it is impossible for a scientist to feel falsely assured that he has discovered all the secrets of nature ... and not just that, but that he has no certain proof that what he has discovered in his research is correct, [because] a colleague or someone in a future generation may come and contradict his scholarly construction, so it is not possible for the person engaged in scholarly Torah study to be certain about his interpretation and to confirm all the advances he has tried to make and investigated, and to claim that he has confirmed them all. Furthermore, there is never proof that his explanation reflects the true meaning of the Torah. Nevertheless, it behooves us to attempt to do all that we have the ability to do.” It seems that the Naziv holds that Torah scholars and natural scientists share a common stance, namely there is no certainty in the outcome of their respective undertakings. This humbling realization of the nature of human pursuit (be it the most noble and worthy), should not be considered an impediment, but rather a liberating idea that should energize the respective scholar to work even harder so that he will flourish in his endeavor.

## CONCLUSION

There is no unique Jewish perspective on evolution, as there should not be a singular Jewish position on any other theoretical scientific issue. As a reflection of their wide interests beyond Halakha, and as intellectually curious and educated members of their respective societies, rabbis, throughout history, maintained diverse opinions on scientific matters deeply rooted in their times and environment. Yet, even the most authoritative of our rabbis rarely hold the opinion that their views on scientific matters reflect the unified view of Judaism.

The Darwinian evolution theory in its current synthesis remains central to the enterprise of biology today. After 150 years of the most intense analysis, debate, and critical testing, the theory of evolution stands as strong as ever with thousands of facts as its empirical base. As Peter Medawar eloquently put it “the alternative to thinking in evolutionary terms is not to think at all”. Whether we like it or not, biology simply means evolution.

## References

[1] Steinberg A (2010). The theory of evolution – A Jewish perspective. RMMJ.

[2] Maimonides M Guide of the Perplexed.

[3] Gould SJ, Gould SJ (1994). Evolution as Fact and Theory. Hen's Teeth and Horse's Toes: Further Reflections in Natural History.

[4] Gould SJ (1994). In the mind of the beholder. Natural History.

[5] Pigliucci M, Kaplan J (2000). The fall and rise of Dr Pan-gloss: adaptationism and the Spandrels paper 20 years later. Trends Ecol Evol.

[6] Loike JD, Tendler MD (2006/2007). Molecular genetics, Evolution and Torah Principles. The Torah u-Madda Journal.

[7] Parham P (1994). The rise and fall of great class I genes. Semin Immunol.

[8] Shanks N, Pyles RA (2007). Evolution and medicine: the long reach of “Dr. Darwin”. Philos Ethics Humanit Med.

[9] Gould SJ (1980). The Panda's Thumb: More Reflections in Natural History.

[10] Parham P (1990). Immunology. Some savage cuts in defense. Nature.

[11] Jacob F (1977). Evolution and tinkering. Science.

[12] Stedman HH, Kozyak BW, Nelson A (2004). Myosin gene mutation correlates with anatomical changes in the human lineage. Nature.

[13] Goodman M, Porter CA, Czelusniak J (1998). Toward a phylogenetic classification of Primates based on DNA evidence complemented by fossil evidence. Mol Phylogenet Evol.

[14] Williams RA, Myers P (1994). Pathology of the appendix and its surgical treatment.

[15] Leibowitz Y, Goldman E (1992). Religion and Science in the Middle Ages and in the Modern Era. Judaism, Human Values and the Jewish State.

[16] Aviezer N Knowledge in the Realm of Science and Knowledge in the Realm of Religion. Are they Different?. Jewish Action.

[17] Jacob CO (2005). Facts or Fiction: Review of “Jewish Philosophy: An Historical Introduction” by Norbert M. Samuelson. Janus Head.

[18] Ivry AL (1989). Staub's Gersonides. The Jewish Quarterly Review.

[19] Maimonides M, Pines S (1963). Guide of the Perplexed.

[20] Misnah, Tractate Hullin 9:10.

[21] Babylonian Talmud, Tractate Hullin 45b.

[22] Maimonides M, Pines S (1963). Guide of the Perplexed.

[23] Steinberg A (2006). Change of Nature. Encyclopedia of Jewish Medical Law.

[24] Babylonian Talmud, Tractate Sukah 5a.

[25] Steinberg A (2006). Preface 3: The basic approach toward scientific discoveries. Encyclopedia of Jewish Medical Law.

[26] Bernard C, Copley Greene H (1927). An Introduction to the Study of Experimental Medicine; 1865.

[27] Babylonian Talmud, Tractate Nedarim 3a.

[28] Maimonides M, Friedlander M (1940). Guide for the Perplexed.

[29] Leibowitz Y, Goldman E (1992). A historical thinkers in Judaism. Judaism, Human Values, and the Jewish State.

